# Editorial: Dry eye disease syndrome, volume II

**DOI:** 10.3389/fmed.2024.1520176

**Published:** 2024-11-21

**Authors:** Alfredo Domínguez-López, Alejandro Navas, Enrique O. Graue-Hernández, Victor L. Perez, Yonathan Garfias

**Affiliations:** ^1^Cell and Tissue Biology and Cornea Departments, Instituto de Oftalmología Fundación de Asistencia Privada Conde de Valenciana, I.A.P, Mexico City, Mexico; ^2^Department of Biochemistry, Faculty of Medicine, Universidad Nacional Autónoma de México, Mexico City, Mexico; ^3^Department of Ophthalmology, School of Medicine, Duke University, Durham, NC, United States

**Keywords:** dry eye disease, corneal inflammation, pathophysiology, diagnosis, treatment

Dry eye disease (DED) is a highly prevalent and multifactorial condition of the ocular surface that significantly affects the quality of life of patients. This Research Topic, contains interesting studies that expand our knowledge of the pathophysiology, diagnosis and treatment of DED.

Several reports indicate a significant association between depression and DED. In this Research Topic, Lee et al. conducted a retrospective case-control study using data from the Taiwan BioBank. They found that the incidence of DED was significantly higher in the depressed population. In addition, patients with both depression and DED had a higher prevalence of sedentary lifestyle and chronic pain than patients without DED. Highlighting the importance of studying lifestyle features in DED population. Morphological alterations in the meibomian glands have been studied in detail in patients with DED. Srivastav et al. studied the distribution of meibomian gland morphology in different age groups in healthy individuals and explored possible age-related changes. The authors identified morphological characteristics of meibomian glands that are considered abnormal in healthy individuals. The findings highlight the importance of evaluating the frequency of all morphological changes, rather than determining their presence or absence, for a more accurate diagnosis. Pflugfelder et al. investigated the difference between the software-detected non-invasive tear break-up time (NIBUT) and the traditional clinical method of fluorescein break-up time (FBUT). Their results showed that software-detected NIBUT is more sensitive in detecting tear film disruptions and capturing patterns missed by traditional methods, and may be more useful in differentiating certain tear disorders.

Inflammation plays a central role in the pathogenesis of the corneal damage and nerve sensitization that is developed in DED. In an interesting study, Alam et al. analyzed the expression profile of corneal immune cells using single-cell RNA sequencing in a desiccating stress dry eye model. They demonstrated that desiccating stress triggered the recruitment of monocytes and their subsequent differentiation into macrophages. In addition, histologic analysis identified the presence of the macrophages near the nerve plexus of the corneal epithelium, suggesting their contribution to the corneal sensorineural changes in DED.

The efficacy of low-dose honey as an ophthalmic formulation in the treatment of DED was investigated by Sanie-Jahromi et al. The authors first found that 1% of honey-supplemented medium (HSM) reduced inflammatory gene expression in stromal keratocytes. In addition, 1% HSM exhibited antibacterial properties against *Staphylococcus aureus* and *Staphylococcus epidermidis*. This study also showed that the use of 1% honey eye drops improved dry eye symptoms and tear film stability in DED patients, suggesting its efficacy as a treatment option. Sánchez-González et al. compared the efficacy of eye drops containing crosslinked hyaluronic acid (CHA) with that of standard hyaluronic acid (HA) in the treatment of DED. They observed that while both treatments improved symptoms, the CHA group showed a significant improvement in tear film stability and patient comfort. These results suggest that CHA-containing eye drops may be more effective than standard HA eye drops in the treatment of DED.

The reader will also find a review by Lin et al. regarding the questionnaires and assessment tools used to evaluate the impact of DED treatments on quality of life of patients. The authors compiled over 200 articles that emphasize the importance of patient-reported outcomes in evaluating the impact of DED treatments. In addition, this review suggests a holistic approach that considers both improvement in objective symptoms and patient wellbeing for comprehensive treatment strategies.

These articles highlight important contributions to the development of new diagnostic tools and treatment strategies for DED. We are convinced that this second volume will be of great interest, as it offers new perspectives for the management of patients with dry eye. Finally, we would like to thank all authors, institutions, reviewers and editors for their valuable time and effort in preparing this Research Topic *Dry Eye Disease Syndrome – Volume II*.

